# A Unidirectional Transition from Migratory to Perivascular Macrophage Is Required for Tumor Cell Intravasation

**DOI:** 10.1016/j.celrep.2018.04.007

**Published:** 2018-05-02

**Authors:** Esther N. Arwert, Allison S. Harney, David Entenberg, Yarong Wang, Erik Sahai, Jeffrey W. Pollard, John S. Condeelis

**Affiliations:** 1 Tumour Cell Biology Laboratory, Francis Crick Institute, London, UK; 2 Gruss-Lipper Biophotonics Center and the Integrated Imaging Program, Albert Einstein College of Medicine, New York, NY, USA; 3 MRC Centre for Reproductive Health, Queen’s Medical Research Institute, The University of Edinburgh, Edinburgh, UK; 4 Department of Developmental and Molecular Biology, Albert Einstein College of Medicine, New York, NY 10461, USA

**Keywords:** tumor associated macrophages, TAMs, TGF beta, breast cancer, metastasis, CXCR4, CCR2, TMEM, Mena

## Abstract

Tumor-associated macrophages (TAMs) are critical for tumor metastasis. Two TAM subsets support cancer cell intravasation: migratory macrophages guide cancer cells toward blood vessels, where sessile perivascular macrophages assist their entry into the blood. However, little is known about the inter-relationship between these functionally distinct TAMs or their possible inter-conversion. We show that motile, streaming TAMs are newly arrived monocytes, recruited via CCR2 signaling, that then differentiate into the sessile perivascular macrophages. This unidirectional process is regulated by CXCL12 and CXCR4. Cancer cells induce TGF-β-dependent upregulation of CXCR4 in monocytes, while CXCL12 expressed by perivascular fibroblasts attracts these motile TAMs toward the blood vessels, bringing motile cancer cells with them. Once on the blood vessel, the migratory TAMs differentiate into perivascular macrophages, promoting vascular leakiness and intravasation.

## Highlights

• Motile TAMs turn into sessile perivascular TAMs via a unidirectional process
• TAMs are recruited via CCR2 signaling, and then tumor-derived TGF-β induces CXCR4
• CXCR4-positive TAMs migrate toward CXCL12-expressing perivascular cancer-associated fibroblasts (CAFs)
• Once on the blood vessel, TAMs become sessile and promote cancer cell intravasation

## Graphical Abstract

**Figure:**
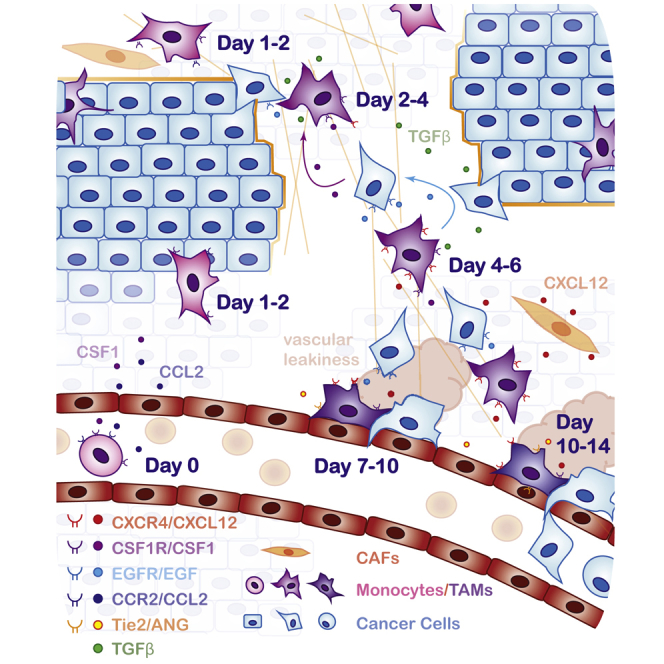

## Teaser

Tumor-associated macrophages (TAMs) are essential for metastasis. Arwert et al. show that, following extravasation, monocytes initially become motile TAMs. Tumor-derived TGF-β then induces CXCR4 on TAMs, stimulating them to migrate toward CXCL12-expressing perivascular fibroblasts. Once adjacent to blood vessels, TAMs differentiate into metastasis-assisting perivascular TAMs.

## Introduction

The diverse functions performed by tumor-associated macrophages (TAMs) are attributed to their specialization into subtypes (Broz et al., 2014, Franklin et al., 2014, Harney et al., 2015, Laoui et al., 2014, Qian and Pollard, 2010), including anti-tumor pro-inflammatory M1 macrophages and pro-tumor immune suppressive or wound healing M2 macrophages. However, the diversity of macrophage types in different tissues and cancers indicates that this is an oversimplification (Lewis et al., 2016). Intravital microscopy has revealed different TAM behaviors linked to their location, including migration-associated streaming and perivascular populations (Broz et al., 2014, Harney et al., 2015, Patsialou et al., 2013, Engelhardt et al., 2012). Tumor cells migrating in streams with TAMs move at higher speeds, in a more direct route, and from greater distances toward blood vessels than tumor cells migrating without TAMs (Leung et al., 2017, Patsialou et al., 2013, Wyckoff et al., 2007). This behavior is enabled by a paracrine loop involving colony-stimulating factor 1 (CSF1) production by cancer cells, epidermal growth factor (EGF) production by TAMs, and release of hepatocyte growth factor (HGF) from endothelial cells (Leung et al., 2017, Patsialou et al., 2009, Wyckoff et al., 2004, Wyckoff et al., 2007). Perivascular macrophages are found in structures called TMEM (tumor microenvironments of metastasis), defined as a macrophage, a Mena (Mammalian Enabled)-overexpressing tumor cell, and an endothelial cell in direct contact (Harney et al., 2015, Pignatelli et al., 2014, Robinson et al., 2009, Rohan et al., 2014). TMEM are responsible for vascular endothelial growth factor A (VEGFA)-driven transient vascular leakiness and tumor cell intravasation and predict distant metastatic disease in breast cancer patients (Harney et al., 2015, Rohan et al., 2014, Sparano et al., 2017).

Despite these advances, the temporal aspects of macrophage subtype specification within primary tumors and the possibility of inter-conversion among subtypes remain largely unexplored. To learn more about these processes, we applied a range of temporally controlled perturbations of TAM populations in the MMTV-PyMT mouse model of breast cancer (Lin et al., 2003).

## Results and Discussion

### Monocyte Labeling Reveals Distinct Temporal and Functional Properties of TAM Subsets

**Figure 1:**
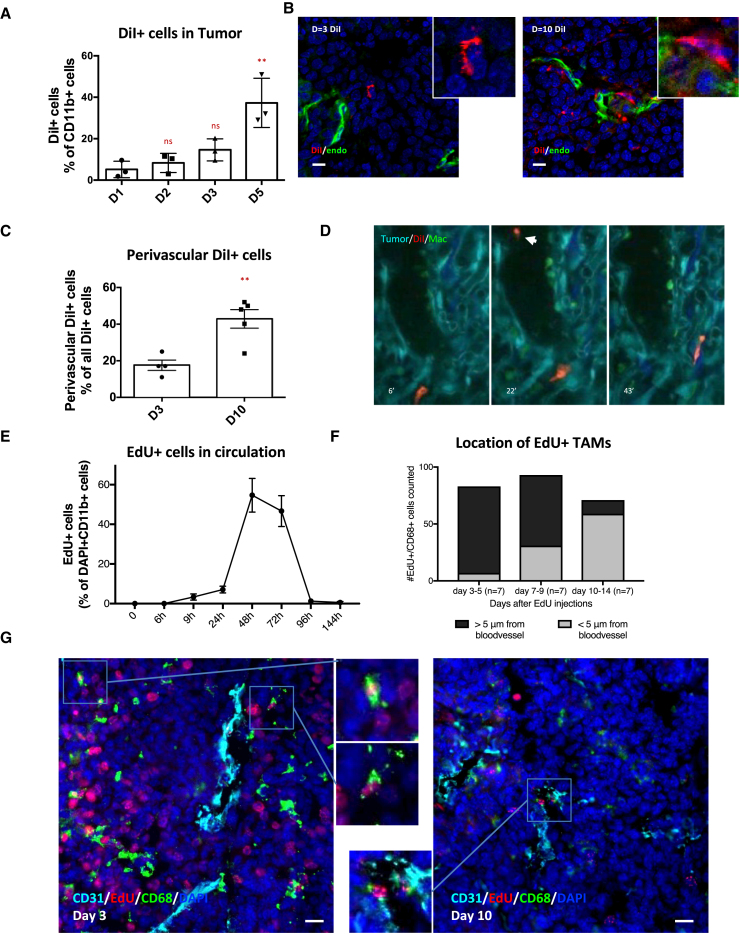
Newly Arriving Monocytes Become Perivascular Macrophages (A) Flow cytometry quantification of the proportion of DiI+ CD11b+ cells in the tumor, measured different days after DiI liposome delivery. (B) Immunofluorescence (IF) of a PyMT tumor at different days after DiI liposome injection, showing cells that ingested DiI (red), endothelial cells (green), and the nuclear counter stain DAPI (blue). Scale bar is 10 μm. Inserts show magnification of one of the cells from the image. (C) Quantification of IF staining showing the proportion of perivascular DiI+ cells. (D) Still frames from Video S1 showing a DiI-labeled macrophage (red) among other macrophages (green) in a tumor (cyan) with collagen fibers (dark blue). The arrowhead indicates a labeled monocyte in the blood stream. (E) Quantification of EdU+ cells in blood smears at different times after EdU injection (n = 3). (F) Quantification of the location of the absolute number of CD68+/EdU+ TAMs, based on IF images as seen in (F) at different times after EdU administration. Per time point, seven PyMT mice were analyzed and the number of CD68+/EdU+ cells analyzed were as follows: days 3–5, 84 cells; days 7–8, 63 cells; and days 10–14, 72 cells. (G) IF imaging of PyMT tumor sections at different days after EdU injection. TAMs are stained for CD68 (green), blood vessels are stained for CD31 (cyan), with DAPI (blue), and EdU+ nuclei indicate cells that were in S-phage at the time of EdU administration (red). Scale bar is 30 μm. Chi-square analysis comparing perivascular versus non-perivascular macrophages. p < 0.0001. Data show mean ± SEM, and each data point represents an individual animal (in A and C).

Clodronate liposomes target phagocytic cells and can deplete monocyte and macrophages (Buiting et al., 1996, Qian et al., 2011, Sunderkötter et al., 2004). In previous studies, we observed a reduction in circulating tumor cells (CTCs) in the PyMT model after clodronate liposome treatment (Patsialou et al., 2013, Roussos et al., 2011). We reconfirmed this, and to our surprise, the reduction in CTCs persisted a week after clodronate treatment, even though liposomes are cleared from the blood within minutes (Figures S1A and S1B) (Buiting et al., 1996). These data argue that TAM function is perturbed for a considerable period following transient clodronate treatment. We therefore set out to track the dynamics of TAMs. To visualize TAMs, we used liposomes loaded with the fluorescent dye 1′-dioctadecyl-3,3,3′3′-tetramethylindocarbocyanine perchlorate (DiI) in tumor-bearing mice (Figures S1A–S1H). As expected, the myeloid cells in the spleen and liver were effectively labeled, but surprisingly, only 3% of myeloid cells in PyMT tumors were labeled after 24 hr; however, this number increased steadily over several days (Figures 1A and S1C–S1F). Similar to the TAMs, monocytes found in tumor blood vessels were not effectively labeled after 24 hr, but this increased after 48 hr (Figure S1G). Staining of tumor sections revealed similar results (Figures 1B and S1C). Moreover, at early time points after DiI liposome injection, only 17% of the DiI+ cells detected inside the tumor were in direct contact with a blood vessel (Figures 1B, 1C, S1H, and S1I). In contrast, 10 days after DiI treatment, 43% of DiI+ cells were in direct contact with blood vessels (Figures 1B and 1C) and more DiI+ cells were found within the tumor compared with earlier time points (Figures S1H and S1I). These data suggest monocyte lineages become labeled with DiI liposomes in hematopoietic tissues and then transit via the blood to tumors, where they gradually accumulate at perivascular sites.

We tracked the behavior of recently arrived DiI-labeled monocytes entering the tumor using MacGreen mice, engineered to have EGFP+ve colony-stimulating factor 1 receptor (Csf1r)-expressing macrophages (Sasmono et al., 2003). Imaging of MacGreen PyMT mice revealed that non-perivascular TAMs (>1 cell diameter from a blood vessel) were in collagen-rich stromal areas at the tumor edge (purple outlines in Figure S2A), with few macrophages found within tumor cell nests (yellow outline in Figure S2A). Non-perivascular (stromal) TAMs exhibited a different distribution of velocities compared to perivascular TAMs, with a distinct population of EGFP+ stromal TAMs having a higher velocity than that of any EGFP+ perivascular TAMs (orange outlines in Figure S2A) (Figures S2A–S2C; Videos S1 and S2) (Harney et al., 2015), thus confirming their distinct phenotype. Intravital imaging 48 hr after DiI injection into MacGreen PyMT mice revealed DiI+ cells in the blood and low numbers of DiI+/GFP+ cells within the tumor (Figure 1D; Video S1). The few DiI+/GFP+ TAMs were often motile and found in stromal areas (Videos S1 and S2), suggesting that newly arriving monocytes are migratory and reside in stromal areas rich in collagen and away from vessels. The inhibitory effect of clodronate liposomes on intravasation after 48 hr is likely due to depletion of recently arrived monocytes involved in streaming migration (Figure S1B) (Patsialou et al., 2013).

### Post-mitotic Monocytes Transition through Non-perivascular Regions before Becoming Perivascular TAMs

To track monocyte subtypes in a non-biased way, we used 5-ethynyl-2′-deoxyuridine (EdU) to label rapidly turning over bone marrow and splenic monocytes (Cheraghali et al., 1994). We dosed tumor-bearing PyMT mice twice with EdU (2.5 hr apart) and harvested tumors after 9 hr (Figure S2D). We did not find any EdU+/CD45+ leukocytes in the blood stream (Figure 1E) but saw clear EdU labeling of tumor cells and CD11b+ cells in bone marrow (Figure S2E). Few CD68+ TAMs in the tumor were EdU+ at the 9 hr time point (<0.1%), suggesting that most CD68+ TAMs in the MMTV-PyMT system are non-proliferative and enabling us to track post-mitotic EdU+ monocytes coming from the bone marrow or the spleen by immunofluorescence (IF) (Figure S2E). The number of EdU+ cells in the blood peaked ∼48 hr, and almost no positive cells were observed by 96 hr, giving us a defined labeled population to track (Figure 1E). After 3 days, EdU+/CD68+ TAMs were predominantly non-perivascular (Figures 1F and 1G) (note the high levels of EdU labeling of tumor cells). However, almost 40% of EdU+/CD68+ TAMs were perivascular after 7 days (Figures 1F and 1G), and this increased to ∼80% at 10 days. The continuing increase in perivascular TAMs between 7 and 10 days, even though no EdU-labeled monocytes were present in the blood, excludes the possibility of perivascular TAMs being recruited directly from the blood.

### Transition from Monocyte into Functional Perivascular TAM Requires 14 Days

**Figure 2:**
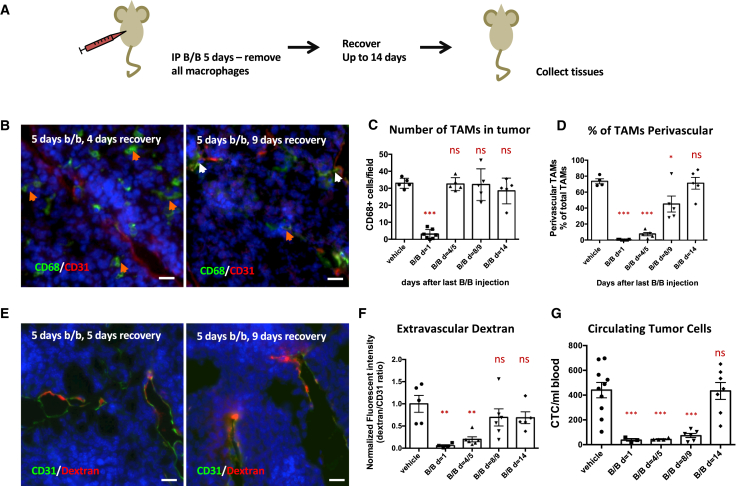
TAM Numbers Quickly Recover after Depletion but Are Initially Non-perivascular and Differentiate into Functional Perivascular TAMs over Time (A) Schematic overview of experiments. (B) IF imaging of PyMT tumor sections at different days after final B/B treatment. Orange arrows show non-perivascular TAMs, while white arrows show perivascular TAMs. TAMs are stained for CD68 (green), blood vessels are stained for CD31 (red), and nuclei are stained with DAPI (blue). Scale bar is 20 μm. (C and D) Quantification of the number of CD68-positive cells found in the tumor tissue (C) and their location in relation to the vasculature (D) at different time points after final B/B treatment. (E) IF imaging of orthotopic PyMT tumor sections at different days after final B/B treatment. Blood vessels are stained with streptavidin against CD31-biotin injected 5 min before sacrifice (green) and dextran (red), and nuclei are stained with DAPI (blue). Scale bar is 20 μm. (F and G) Quantification of extravascular 155 kDa dextran TMR as a measurement of vascular leakiness (F) and number of CTCs found per milliliter of blood (G) different days after the last B/B injection. Data show mean ± SEM, and each data point represents an individual animal (in C, D, F, and G).

To further test whether streaming TAMs transition into stationary perivascular TAMs, we transiently depleted all macrophages using the macrophage Fas-induced apoptosis (MaFIA) mouse model with orthotopically implanted PyMT tumors. In this model, 5 days of treatment with the small molecule AP20187 (also known as the B/B homodimer) effectively removes >90% of TAMs by apoptosis (Clifford et al., 2013, Harney et al., 2015). We followed the repopulation of TAMs and their spatial location by IF (Figures 2A and S3). Although the number of CD68+ TAMs returned to control levels within 4 days after termination of B/B treatment (Figures 2B, 2C, and S3), the TAMs were rarely found in contact with blood vessels stained with either CD31 or endomucin. It took up to 8 days after the end of B/B treatment for the number of perivascular CD68+ TAMs return to control levels (Figures 2B and 2D). We additionally characterized the expression of markers linked to perivascular macrophage biology: VEGFA, CD206, and LYVE-1 (Figures S4A–S4C and S5A–S5D) (Harney et al., 2015, Zeisel et al., 2015). CD206 stained both perivascular and non-perivascular TAMs at early time points and, similar to CD68, showed the same transition to predominantly perivascular staining later. We observed low levels of VEGFA staining in CD68+ cells, but these showed a similar trend of accumulation in perivascular regions 7 days after B/B treatment. We did not observe LYVE-1 staining in any TAMs (Figure S5D).

We also tracked the recovery of perivascular TAM functionality after B/B treatment by examining vessel leakiness and CTC numbers. Restoration of vessel leakiness to control levels coincided with the return of perivascular TAMs 8 days after the last B/B treatment (Figures 2C, 2E, and 2F). However, the number of CTCs took 14 days to recover after B/B treatment (Figure 2G), indicating that perivascular leakiness is restored quickly after TAMs contact the vasculature but that the reestablishment of TAM functions that aid tumor cell intravasation takes longer.

### CCR2 Is Required for Monocyte Recruitment

**Figure 3:**
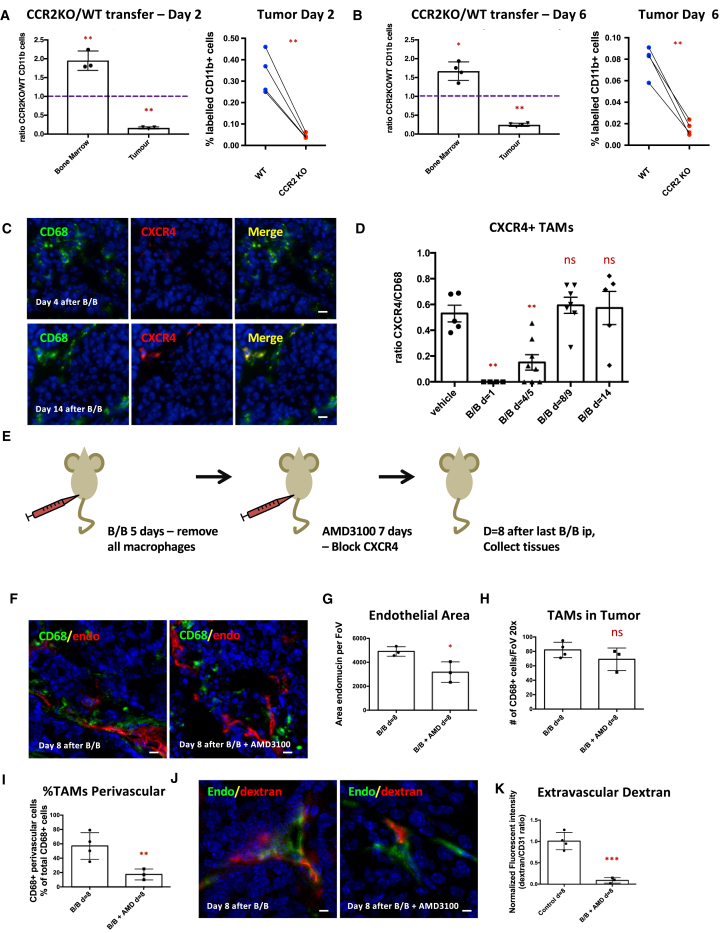
Recruitment of TAMs into the Tumor Depends on CCR2, while Recruitment of Perivascular TAMs to the Blood Vessels Depends on CXCR4 Signaling (A and B) FACS analysis of the ratio of CCR2 KO/WT monocytes found in bone marrow or tumor 2 days (A) or 6 days (B) after adoptive transfer (n = 4 mice per time point). The purple line indicates the ratio of monocytes upon injection. (C) IF imaging of PyMT tumor sections at different days after final B/B treatment. TAMs are visualized with CD68 (green) and CXCR4 (red), and nuclei are stained with DAPI (blue). Scale bar is 10 μm. (D) Quantification of the number of CXCR4+ TAMS found at different times after final B/B injection. (E) Schematic overview of experiments with AMD3100/CXCR4 inhibitor. (F) IF imaging of PyMT tumor sections after 5 days of B/B treatment, followed by 7 days of AMD3100 compared to controls. TAMs are visualized with CD68 (green), vasculature is visualized with endomucin (red), and nuclei are stained with DAPI (blue). Scale bar is 20 μm. (G–I) Quantification of the area blood vessels per field of view (FoV) (G), macrophages per FoV (H), and perivascular macrophages per FoV (I). (J) IF imaging of PyMT tumor after 5 days of B/B treatment, followed by 7 days of AMD3100. Blood vessels are stained with streptavidin against CD31-biotin injected 5 min before sacrifice (green) or extravascular dextran (red), and nuclei are stained with DAPI (blue). Scale bar is 20 μm. (K) Quantification of extravascular 155 kDa dextran TMR as a measurement of vascular leakiness. Data show mean ± SEM, and each data point represents an individual animal (in A, B, D, G–I, and K).

Having established that circulating monocytes transition through a migratory phase into sessile perivascular macrophages, we sought to identify the molecular regulators of these steps. We tested whether CCR2, a receptor that mediates monocyte chemotaxis, was required for the initial recruitment of monocytes. A mixture of differentially labeled CCR2 wild-type and −/− bone marrow-derived monocytes was adoptively transferred into tumor-bearing mice. The proportion of wild-type to CCR2 −/− CD11b+ cells was tested 2 and 6 days later (Figure S6). CCR2 −/− cells were dramatically under-represented in the tumor, despite their ability to colonize the bone marrow (Figures 3A and 3B).

### CXCR4 Signaling Regulates TAM Homing to Blood Vessels

CXCR4 expression has been associated with TAM recruitment and differentiation (Hughes et al., 2015). Therefore, we studied the CXCR4 expression levels in CD68+ TAMs at different time points after B/B treatment. Only a small percentage of CD68+ macrophages expressed CXCR4 4 days after arrival in the tumor (Figures 3C and 3D), suggesting that CXCR4 is not important for their initial recruitment. To test whether the CXCR4/CXCL12 axis is required for migratory macrophages to become functional perivascular TAMs, we used the CXCR4 antagonist AMD3100. We depleted TAMs with B/B treatment, followed by a 7-day, twice-daily AMD3100 treatment during the recovery phase (Figure 3E). As previously noted, CXCR4 blockade reduced the vascular area in tumors (Figures 3F and 3G) (Hughes et al., 2015), but the number of CD68+ TAMs was similar to controls (Figures 3F and 3H). The percentage of TAMs directly interacting with blood vessels was greatly reduced in the AMD3100-treated tumors (Figures 3F and 3I), and this correlated with a reduction in vascular leakiness (Figures 3J and 3K).

**Figure 4:**
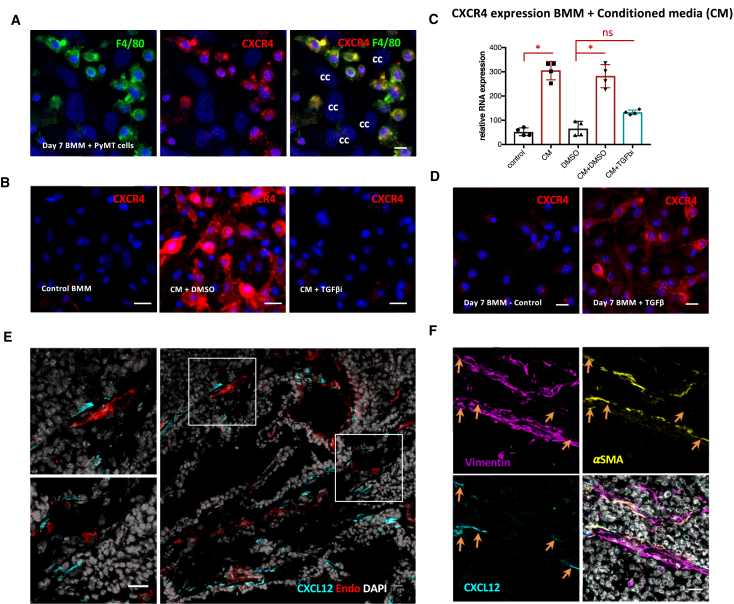
Cancer Cell-Derived TGF-β Upregulates CXCR4 on BMMs, and CXCL12 Is Produced by Fibroblasts (A) IF of BMMs co-cultured with PyMT cancer cells. Cells are stained for F4/80 (green), CXCR4 (red), and DAPI (blue). cc, cancer cells. Scale bar is 10 μm. (B) IF of BMMs cultured in BMM media with or without the addition of PyMT cancer cell conditioned media (CM) and transforming growth factor β receptor (TGF-βR) inhibitor. BMMs are stained with CXCR4 (red) and DAPI (blue). Scale bar is 10 μm. (C) qPCR analysis for CXCR4 RNA expression performed on the RNA isolated from BMMs treated with PyMT cancer cell CM with (blue) or without (burgundy) TGF-βR inhibitor (TGF-βi). (D) IF of BMMs cultured in BMM media without or with the addition of TGF-β (2 ng/mL). BMMs are stained for CXCR4 (red) and DAPI (blue). Scale bar is 10 μm. (E) IF of PyMT sections stained with CXCL12 (cyan), endomucin (red), and DAPI (gray). Scale bar is 20 μm. (F) IF of a PyMT tumor section stained with vimentin (magenta), α-smooth muscle actin (αSMA, yellow), CXCL12 (cyan), and DAPI (gray). Scale bar is 20 μm. Data show mean ± SEM, and each data point represents an individual animal (in C, D, F, and G).

The data outlined earlier establish CXCR4/CXCL12 signaling in directing TAMs toward the blood vessels; however, they do not explain what upregulates CXCR4 or identify the source of CXCL12. We tested whether tumor-derived factors could upregulate CXCR4 by co-culturing bone marrow-derived macrophages (BMMs) with PyMT cancer cells. This co-culture resulted in strong CXCR4 expression in F4/80-positive BMMs (Figure 4A), and exposure of BMMs to cancer cell conditioned media triggered a marked upregulation of CXCR4 in macrophages (Figures 4B and 4C) Crucially, inhibition of transforming growth factor β (TGF-β) signaling blocked the induction of CXCR4 mRNA and protein by cancer cell conditioned media (Figures 4B and 4C), and TGF-β was sufficient to induce CXCR4 (Figure 4D) (Chen et al., 2005).

Immunofluorescence analysis of tumor sections revealed that CXCL12 is expressed by elongated cells frequently adjacent to blood vessels (Figure 4E). Co-staining demonstrated that three-fourths of CXCL12-expressing cells were positive for the generic fibroblastic marker vimentin and roughly 10% were positive for the more specific cancer-associated fibroblast marker α-smooth muscle actin (αSMA) (Figures 4E, 4F, and S7A–S7C). There was no overlap with either the endothelial marker endomucin or the pericyte marker desmin (Figures 4E, 4F, and S7D). These data indicate that CXCL12 is expressed by stromal fibroblasts proximal to blood vessels and explain the recruitment of TAMs to perivascular regions following the TGF-β-driven induction of CXCR4. Other studies have shown that ANG2/Tie2 blockage results in a phenotype similar to the one we observe following CXCR4 blockade with reduced vascular density and failure of TAMs to attach to the blood vessels (Harney et al., 2017, Mazzieri et al., 2011). In addition, our results agree with previous work in which AMD3100 treatment led to a preferential reduction in perivascular macrophages (Welford et al., 2011). We propose that CXCR4/CXCL12 is important for migration to blood vessels, while Tie2 is required for attachment to the endothelial cells and maturation into a functional perivascular TAM regulating vascular leakiness and cancer cell intravasation (Harney et al., 2015).

To conclude, we propose that monocytes, recruited via CCR2 signaling, initially become motile streaming TAMs before a TGF-β-dependent conversion into CXCR4-expressing macrophages. These TAMs are then recruited to become sessile perivascular TAMs by CXCL12. This unidirectional differentiation process takes 10–14 days. This argues against the view that once educated, the macrophage phenotype does not change, and it refutes the opposing idea that unrestricted inter-conversion between macrophage states is possible. Instead, we document a surprisingly stereotypic and unidirectional conversion between macrophage states. Single-cell RNA sequencing (RNA-seq) analysis suggests that a similar situation applies in human breast cancer (Azizi et al., 2017). In the future, it will be interesting to explore this dynamic in the context of chemotherapy (Hughes et al., 2015, Karagiannis et al., 2017) and other perturbations, which in some cases may trigger local proliferation of macrophages (Franklin et al., 2014). An improved understanding of the lineage and temporal dynamics of different TAM subsets will be important for optimizing the targeting of TAMs for therapeutic benefit.

## Experimental Procedures

### Mice

All mice studies were carried out in accordance with NIH regulation (US) or UK Home regulation (UK). Procedures were approved by the Albert Einstein College of Medicine Animal Care (animal use protocol 20130909) and by the Francis Crick Institute Biological Ethics Committee (project license 70/8380). MMTV-PyMTmice were maintained on a susceptibility to Friend leukemia virus B/NIH (FVB/N) background and were crossed with MMTV-Cre and lox-stop-lox (LSL)-eGFP or with a co-integrated allele FVB/N MMTV.improvedCre.LSL enhanced Cerulean Fluorescent Protein (eCFP)_jwp_ mice to develop mice with green or blue mammary gland tumors. MacGreen mice (Sasmono et al., 2003) were crossed with PyMT FVB mice to develop MacGreen-PyMT mice (Ahmed et al., 2002). Age-matched females were used in experiments when they were around 12–14 weeks old. MaFIA mice, known as C57BL/6 Tg (Csf1r-EGFP NGFR/FKBP1A/TNFRSF6)2Bck/J, were obtained from The Jackson Laboratory. All experiments with MaFIA mice were performed with implantation of orthotopic C57BL/6 PyMT tumors in MaFIA mice. The tumors were developed by implantation of tumor pieces (2 × 2 mm) of late-stage spontaneous C57BL/6 PyMT tumors into the mammary fat pad of 5- to 7-week-old female MaFIA mice. Typically, after 6–7 weeks, single PyMT tumors appeared. Experiments were typically performed on 0.6–0.8 cm tumors. Multiphoton intravital microscopy was performed as previously described (Harney et al., 2015). Under general anesthesia, the mouse was placed on a heated microscope stage, with the surgically exposed tumor placed onto a cover glass. Imaging was performed using a custom-built 2-laser multiphoton microscope (Entenberg et al., 2011).

### FACS Analysis of Tumors, Spleen, and Blood Samples

Tumor or spleen samples were prepared by tissue digestion using Liberase and Dispase (Roche), combined with red blood cell (RBC) lysis (eBioscience) as previously reported (Qian et al., 2011). Blood cells were isolated by cardiac puncture, followed by RBC lysis. Cells were blocked with an anti-mouse CD16/CD32 fragment crystallizable (Fc) blocking antibody for 10 min before antibody staining (BD Biosciences). Gating was used to exclude dead cells, cell doublets, and clusters. In certain experiments, mice were injected with CD45-fluorescein isothiocyanate (FITC) (eBioscience) 2–3 min before sacrifice to label all immune cells in the blood, but not in the tissues, at the time of death to exclude those cells from the tissue analysis, as well as to measure immune cells inside the blood at the time of death (Tagliani et al., 2011). Data were analyzed with FlowJo software (Tree Star).

### Liposome and Edu Treatment

The liposomes clodronate, control, and DiI (1′-dioctadecyl-3,3,3′3′-tetramethylindocarbocyanine perchlorate) (Clodrosome) were injected into the tail vein at 5 mL/kg (Buiting et al., 1996, Sunderkötter et al., 2004) in two doses 24 hr apart in age-matched, tumor-bearing PyMT females around 12–14 weeks of age. Either 48 hr or 7 days after the first clodronate liposome injection, CTCs were isolated (as described later). After the last DiI liposome injections, tissue samples were isolated at various time points (as described earlier) for IF staining (as described later) or fluorescence-activated cell sorting (FACS) analysis (as described earlier). EdU (5-ethynyl-2′-deoxyuridine) was dissolved in a saline solution and injected via tail vein twice at 40 mg/kg, with a 2.5 hr rest between injections (Cheraghali et al., 1994).

### Blood and Bone Marrow Smears

Bone marrow cells were isolated from the femur, and a suspension was created in a small volume of PBS and spread into a smear on glass slides. Blood smears were created with blood from the lateral tail vein. The slides were air-dried and subsequently fixed with 100% methanol for 2 min, air-dried, and kept at 4°C until ready to perform the immunofluorescence (IF) protocol described later.

### Macrophage Depletion Studies in MaFIA Mice

10 mg/kg B/B homodimerizer (AP20187, Clontech) diluted in 4% ethanol, 10% PEG-400, and 1.7% Tween 20 or vehicle control was injected intraperitoneally on 5 subsequent days. Treatment was started when tumors were 0.6–0.8 cm (in diameter), and typically, they were not larger than 1.0–1.1 cm by the end of the experiment. AMD3100 was administered at 5 mg/kg twice a day via intraperitoneal injection for 7 days.

### Labeling of Vasculature and Measuring Vascular Leakiness

Measurement of vascular leakiness was performed as previously described (Harney et al., 2015). One hour before the termination of the experiment, 155 kDa- dextran-tetramethylrhodamine (TMR) (Sigma) was injected intravenously (i.v.), and CD31-biotin was injected i.v. 5 min before the end of the experiment, labeling all active blood vessels. Tumors were fixed overnight in 4% paraformaldehyde (PFA), transferred to 30% sucrose, and embedded in optimal cutting temperature (OCT) compound. 8 μm sections were cut, and IF was performed as described later. Extravascular dextran was measured as previously described using ImageJ.

### Immunofluorescence

Cells or tumor sections were fixed and permeabilized with ice-cold acetone (sections) or ethanol (BMMs) for 10 min, blocked for 1 hr with blocking buffer (1% BSA, 5% fetal bovine serum [FBS], and 0.1% fish skin gelatin in PBS-T). Primary antibodies were incubated overnight at 4°C, followed by PBS-T washes and secondary antibody or fluorescently tagged streptavidin (Invitrogen) incubation combined with DAPI for 1 hr at room temperature. EdU was visualized with an EdU click-assay (Molecular Probes) according to the manufacturer’s instructions, followed by the same IF protocol described earlier. Images were acquired using a Zeiss Axio Observer with a 40× objective or a Zeiss LSM 780 confocal microscope with a 20× objective. Images were subsequently imported into ImageJ for analysis.

### Circulating Tumor Cells

Blood was collected from the right ventricle of the heart into a heparin-coated syringe under terminal anesthesia. After RBC lysis (eBioscience), cells were seeded into DMEM/F12 media supplemented with 20% FCS and Pen/Strep, and single tumor cells were counted 7 days after initial plating. Data included here have new data collated with controls previously reported in Harney et al. (2015).

### Adoptive Transfer

Monocytes were isolated by crushing the femur and tibia of female wild-type C57BL/6 or CCR2 knockout (KO) mice, followed by purification with a magnetic-activated cell sorting (MACS) monocyte isolation kit (Miltenyi). Monocytes were labeled with (5(6)-Carboxyfluorescein N-hydroxysuccinimidyl ester (CSFE) or CellTrace Violet (Invitrogen) in PBS, washed, and counted. A 50:50 mixture of CCR2 KO-to-wild-type (WT) monocytes was prepared and injected in a volume of 100 μL of PBS into the tail vein of tumor-bearing C57BL/6 mice with surgically implanted PyMT tumors. A minimum of 5 × 10^6^ monocytes of each subtype were injected. Two or six days after the adoptive transfer, tissues were harvested and analyzed.

### BMMs and Conditioned Media Experiments

Bone marrow cells were isolated from the mouse leg bones (femur and tibia) by flushing with PBS and then differentiated into BMMs with macrophage colony-stimulating factor (MCSF) (10 ng/mL, PeproTech) in BMM media (DMEM/F12, Gibco) supplemented with 10% FBS, Pen/Strep, and 10 ng/mL MCSF). When used for experiments, TGF-β (2 ng/mL, PeproTech) was added after the first media change, typically 2 days after the isolation, for the duration of the experiment. PyMT cancer cell isolates were prepared as previously described (Malanchi et al., 2011). 36 hr were allowed for cancer cells to generate conditioned media (CM), which was then pushed through a 0.4 μm syringe filter (Fisher Scientific). For the treatment of BMMs with CM, 2–3 days after initial bone marrow isolation, non-adhered bone marrow cells were washed away and fresh media diluted 50:50 with filtered CM were added either with or without TGF-β receptor inhibitor (5 μM SB-505124, Sigma). Media were changed every 2 days until the end of the experiment.

### RNA Isolation and qPCR

Cells were collected in RNAprotect reagent (QIAGEN) and kept frozen until RNA extraction using RNeasy mini kits (QIAGEN). The cDNA library was prepared using M-MuLV reverse transcriptase (New England Biolabs), and qPCR was performed using SYBR-green platinum (Invitrogen) assays on the QuantStudio 7 Real-Time PCR systems (Applied Biosystems) with at least two housekeeping genes for normalization.

### Statistical Analysis

Statistical analysis were performed using the chi-square for contingency table test with EdU data, the standard two-tailed Student’s t test for comparing two datasets, and ANOVA followed by Tukey’s or Dunnett’s multiple-comparison post hoc tests for multiple datasets when the sample size was large enough to confirm normality (Shapiro-Wilk). For smaller datasets, Kruskal-Wallis was used followed by Dunn’s multiple-comparison test. To examine the distribution of macrophage motility, the Kolmogorov-Smirnov test was used. All statistical analysis was done using Prism (GraphPad). All graphs show the number of mice indicated as separate data points. Level of significance is indicated with red * (p < 0.05), ** (p < 0.01) or *** (p<0.001).

## Supplemental Information

Document S1. Supplemental Experimental Procedures and Figures S1–S7

Video S1. Example of Csf1r-EGFP Myeloid Cell Behavior, Related to Figure 1 PyMT tumor is shown in light blue, blood vessels are red (Texas Red dextran injection 2MDa), collagen fibers are dark blue and Csf1r-expressing myeloid cells are green.

Video S2. Example Csf1r-EGFP+ Cell Tracking, Related to Figure 1 PyMT tumor is shown in light blue, blood vessels are red (Texas Red dextran injection 2MDa), collagen fibers are dark blue and Csf1r-expressing myeloid cells are green. Different color lines represent individual paths, note perivascular cells do not move away throughout movie therefor the tracking-line will appear as a dot.

Video S3. Example of DiI+ TAMs 2 Days after DiI Injection, Related to Figure 1 DiI is shown in red, collagen in shown in dark blue, tumor in cyan and Csf1r-expressing myeloid cells are green.

Video S4. Example of DiI+ TAMs 2 Days after DiI Injection, Related to Figure 1 DiI is shown in red, collagen in shown in dark blue, tumor in green.

Document S2. Article plus Supplemental Information
