# Supplementary material for: A Unidirectional Transition from Migratory to Perivascular Macrophage Is Required for Tumor Cell Intravasation
Source: Cell Rep. 2018 May 2;23(5):1239–48. doi: 10.1016/j.celrep.2018.04.007 (PMC5946803; doi:10.1016/j.celrep.2018.04.007)
Supplement: Document S1. Supplemental Experimental Procedures and Figures S1–S7 [file mmc1.pdf]

Cell Reports, Volume *23*

Supplemental Information

A Unidirectional Transition from Migratory to Perivascular Macrophage Is Required for Tumor Cell Intravasation

Esther N. Arwert, Allison S. Harney, David Entenberg, Yarong Wang, Erik Sahai, Jeffrey W. Pollard, and John S. Condeelis

Supplemental experimental procedures

Primers (Sigma) used for qPCR:

mCXCR4:

For: 5'-AGCCTGTGGATGGTGGTGTTTC-3', Rev: 5’- CCTTGCTTGATGACTCCCAAAAG -3'

Housekeeping genes:

CPH: For: 5'- ATGGTCAACCCCACCGTG-3', Rev: 5' -TTCTTGCTGTCTTTGGAACTTTGTC - 3',

GAPDH For: 5’-GTGCAGTGCCAGCCTCGTCC-3’, Rev: 5’-GCCACTGCAAATGGCAGCCC-3’

ACTB For: 5’-GGAAGGTGACAGCATTGCTTC-3’, Rev: 5’-GGTCTCAAGTCAGTGTACAGG-3’

Antibodies used for IF & FACS

Antigen	Fluorochrome	Host	Clone/antibody #	Provider	Dilution
CD45	BV510/PE/FITC	Rat mono	30-F11	Biolegend	1:100
CD31	biotin	Rat mono	390	Biolegend	1:100
CD31	AF488	Goat poly	# FAB3628G	R&D systems	1:50
CD68	AF488/594/647	Rat mono	FA-11	Biolegend	1:100
CD11b	FITC/APC	Rat mono	M1/70	eBioscience	1:100
Gr-1	PE-cy7	Rat mono	RB6-8C5	eBioscience	1:100
F4/80	FITC/PE/APC/AF594	Rat mono	BM8	eBioscience	1:100
CD206	AF647	Rat mono	C068C2	Biolegend	1:200
streptavidin	488/555/647	N/A	N/A	Invitrogen	1:800
endomucin	FITC/AF680 or unconjugated	Rat mono	V.7C7	Santa Cruz	1:50/1:200
aSMA	Cy3	Mouse mono	1a4	Sigma	1:200
CXCR4	unconjugated	Rabbit mono	UMB2	Abcam	1:200
CXCR4	unconjugated	Rabbit poly	# H-188	Santa Cruz	1:50
Vimentin	unconjugated	Mouse mono	LN-6	Sigma	1:200
CXCL12	unconjugated	Rabbit poly	# 14-7992-81	Biolegend	1:100

Figure S1 – **Related to Figure 1**

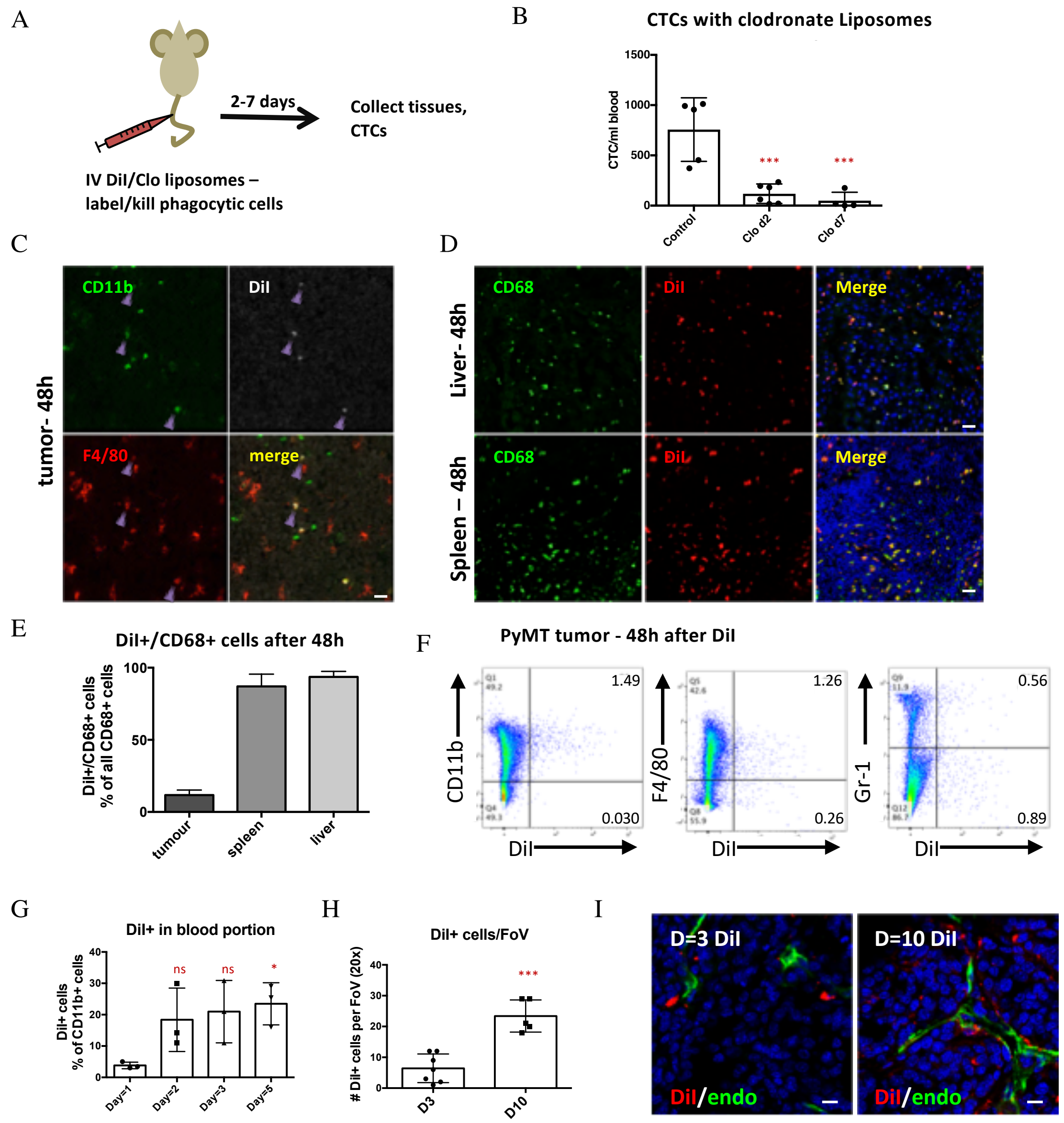

Figure S1 – DiI liposomes and EdU label few cells inside the tumor – related to Figure 1 (A) Schematic overview of experiments with DiI and clodronate liposomes. (B) Number of CTCs found per ml of blood in PyMT mice treated with PBS or clodronate liposomes: two and seven days after treatment. (C) Immunofluorescence (IF) imaging of PyMT tumor sections 48h after DiI liposome injection. TAMs are stained by CD11b (green) and F4/80 (red), a few DiI+ cells (grey) are indicated by an arrowhead. Scale bar is 10µm (D) IF imaging of liver or spleen sections 48h after DiI liposome injection. Macrophages are visualized by CD68-FITC (green), cells with DiI appear in red, nuclear counterstain: DAPI (blue). Scale bar is 20µm (E) Quantification of the proportion of CD68+ macrophages that took up DiI 48h after DiI liposome injection in tumor, spleen and liver. (F) Representative FACS plots gated on Single cells, DAPI- (alive), CD45+ cells showing DiI positive cells on X-axis and CD11b+, F4/80 or Gr-1 on Y-axis. Note that virtually all DiI+ cells are also CD11b+. (G) Quantification of the proportion of CD11b myeloid cells in the blood-portion of the tumor isolated used for FACS analysis that took up DiI at different times after DiI liposome injection. (H) IF imaging of a PyMT tumor at different days after DiI liposome injection, showing cells that ingested DiI (red), endothelial cells (green), nuclear counter stain: DAPI (blue). Scale bar is 10µm. (I) Number of DiI+ cells within a field of view, based on images like (H) and Fig 1B. Data shows mean ±SD, each data point represents an individual animal (C).

Figure S2 – **Related to Figure 1**

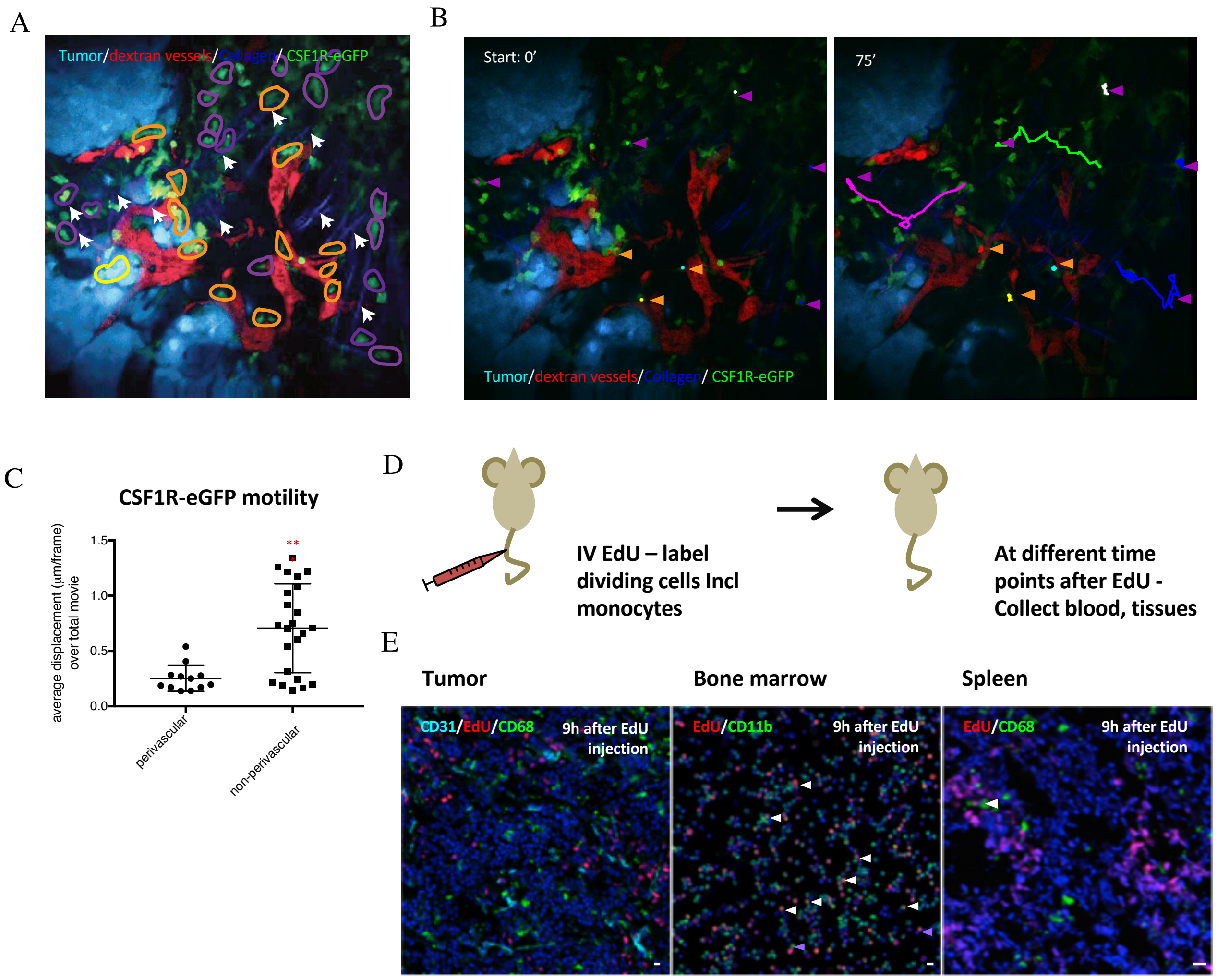

Figure S2 – DiI liposomes and EdU label few cells inside the tumor – related to Figure 1. (A) Still from Movie S1 indicating different types of CSF1R-eGFP niches: within the tumor cell nest (yellow), perivascular (orange) and non-perivascular cells (purple) in stromal, collagen-rich areas (white arrows indicating collagen fibers identified by second harmonic signal. (B) An example of the tracking of CSF1R-eGFP+ cells at start (arrowheads indicating starting points of cells: orange (perivascular) and purple (stromal/non-perivascular) and end of tracking. Different color lines represent individual paths, note perivascular cells do not move away throughout movie therefor the tracking-line will appear as a dot. (C) Speed analysis of CSF1R-eGFP+ cells within PyMT tumors. eGFP+ cells were picked at start of movie based on their location and followed as long as they were visible with ImageJ manual tracking plug-in (n=3 PyMT animals) Data shows mean $\pm$SEM, each data point represents an individual macrophage. (D) Schematic illustration of the EdU labelling experiment design. (E) Fluorescent micrographs of section of tumor (left panel), bone marrow smear (middle panel) and blood smear (right panel). EdU+ cells (red), macrophages are visualized by CD68 (green) and blood vessels by CD31 (cyan). Myeloid cells in the bone marrow are visualized by CD11b (green). White arrowheads indicate a few proliferating CD11b+ in the bone marrow or CD68+ cells in the spleen. Scale bar is 20μm. Note that there are no EdU+/CD68+ cells found in the tumor at this time point (left panel) or in the blood (Fig 1E), while several EdU+/CD11b+ cells are found within the bone marrow and a few EdU+/CD68+ cells in the spleen.

Figure S3 **Related to Figure 2**

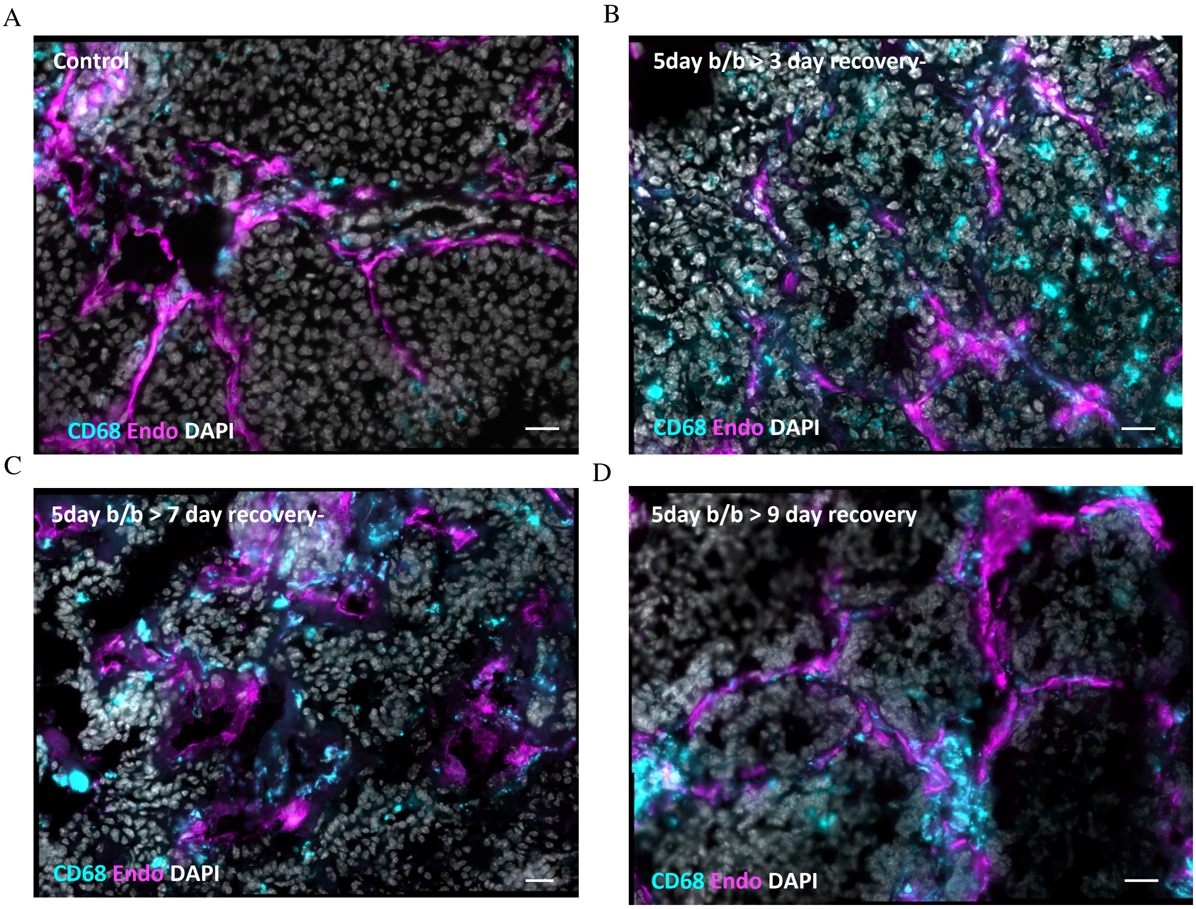

Figure S3 – Additional marker stainings of macrophage depletion experiments – related to Figure 2. (A-D) IF imaging of PyMT tumor sections at different days after final B/B treatment. TAMs are visualized with CD68 (cyan) and endomucin (magenta), nuclei are stained with DAPI (grey), scale bar is 25µm

Figure S4 – **Related to Figure 2**

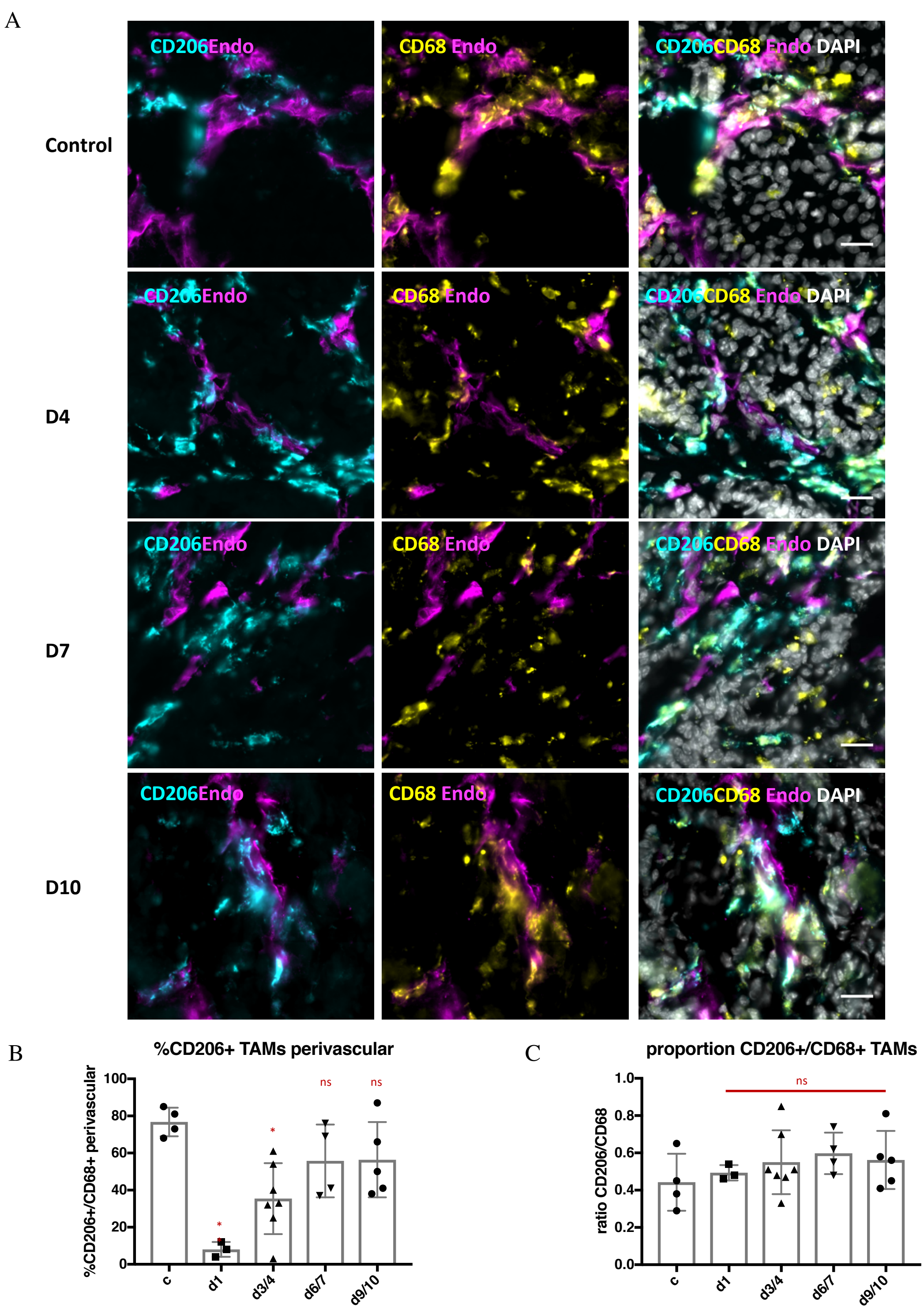

Figure S4 – Additional marker stainings of macrophage depletion experiments – related to Figure 2. (A) IF imaging of MaFIA tumor sections at different times after the final b/b treatment. TAMs are visualized with CD206/MRC1 (cyan), CD68 (yellow) and blood vessels with endomucin (magenta), nuclei are stained with DAPI (grey), scale bar is 25µm. (B Quantification of images as seen in (B) showing the % of perivascular TAMs that are also CD206 positive. (C) Quantification of images as seen in (B) showing the frequency of CD206 positive TAMs.

Figure S5 **– Related to Figure 2**

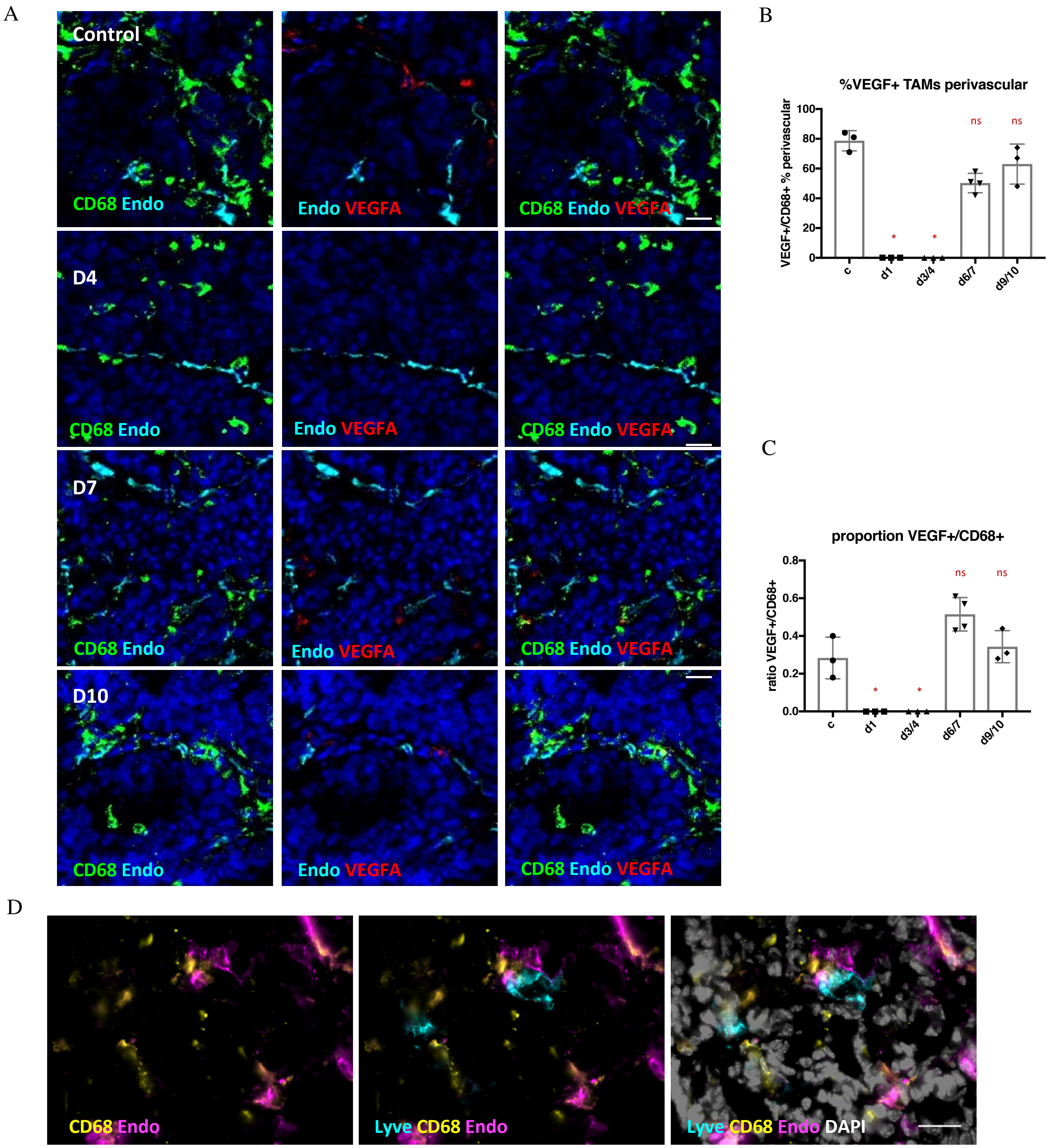

Figure S5– Additional marker stainings of macrophage depletion experiments – related to Figure 2. (A) IF imaging of MaFIA tumor sections at different times after the final b/b treatment. TAMs are visualized with CD68 (green), VEGFA (red) and blood vessels with endomucin (cyan), nuclei are stained with DAPI (blue), scale bar is 25µm. (B) Quantification of images as seen in (E) showing the % of perivascular TAMs that are also VEGFA positive. (C) Quantification of images as seen in (E) showing the frequency of VEGFA positive TAMs. (D) IF imaging of PyMT tumor sections. TAMs are visualized with CD68 (yellow), lymphatic vessels with lyve-1 (cyan) and blood vessels with endomucin (magenta), nuclei are stained with DAPI (grey), scale bar is 25µm

Figure S6 – **Related to Figure 3**

A B

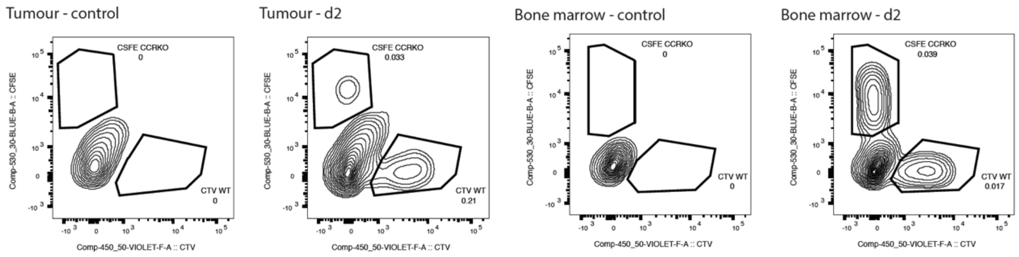

Figure S6 – Exemplar flow cytometry of CCR2 WT and KO adoptive transfer – related to Figure 3. (A, B) Representative logarithmic contour FACS plots used in the analysis of the adoptive transfer experiments with CCR2KO and WT monocytes in tumor (A) or bone marrow (B). Gated on alive, singlets, CD45+, CD11b+ cells. Horizontal axis shows CellTrace Violet, while vertical axis shows CSFE intensity.

Figure S7 – **Related to Figure 4**

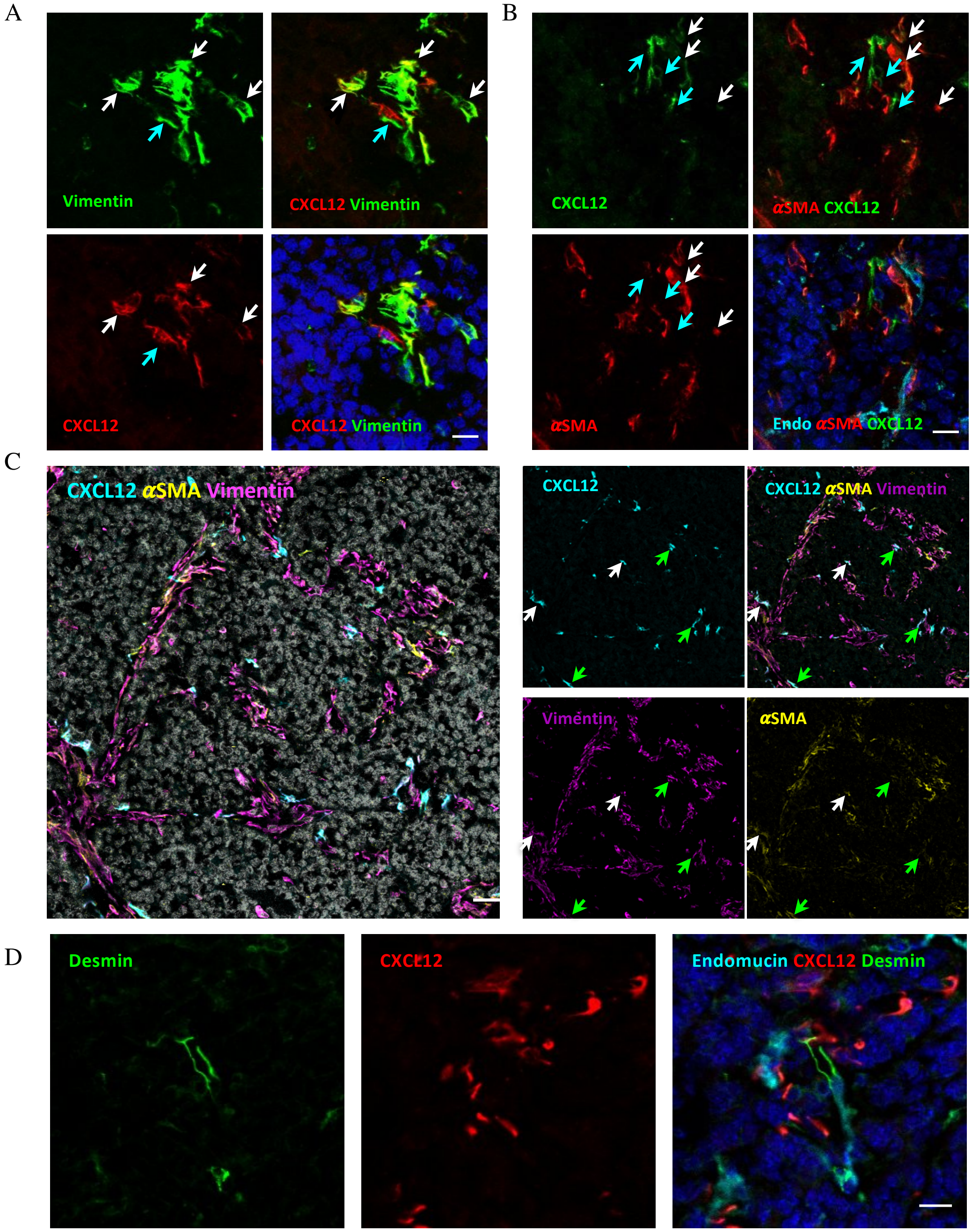

Figure S7 – Stromal fibroblasts, not pericytes are expressing CXCL12 – related to Figure 4. (A) IF imaging of PyMT tumor sections. Fibroblasts are visualized with vimentin (green), CXCL12 (red) and nuclei are stained with DAPI (blue), scale bar is 25µm. White arrows show co-localization of CXCL12 with vimentin, while blue arrows show CXCL12 positive cells that are not vimentin positive. (B) IF imaging of PyMT tumor sections. Fibroblasts are visualized with αSMA (red), CXCL12 (green), endothelial cells with endomucin (cyan) and nuclei are stained with DAPI (blue), scale bar is 25µm. White arrows show a slight co-localization of CXCL12 with αSMA, while blue arrows show CXCL12 positive cells that are not vimentin positive. (C) low powered IF image of PyMT tumor section stained with CXCL12 (cyan), αSMA (yellow) and vimentin (magenta) and DAPI in (grey), white arrows indicate CXCL12 CAFs that are double positive for vimentin and αSMA, while green arrow show CXCL12 CAFs that are single positive for either vimentin or αSMA. Scale bar is 25µm. (D) IF staining of a PyMT section. Pericytes are visualized with Desmin (green) CXCL12 (red) and blood vessels with endomuscin (cyan). Scale bar is 25µm
